# Utility of NT-proBNP as a rule-out test for left ventricular dysfunction in very old people with limiting dyspnoea: the Newcastle 85+ Study

**DOI:** 10.1186/1471-2261-14-128

**Published:** 2014-09-26

**Authors:** Joanna Collerton, Andrew Kingston, Fahad Yousaf, Karen Davies, Antoinette Kenny, Dermot Neely, Carmen Martin-Ruiz, Guy MacGowan, Louise Robinson, Thomas BL Kirkwood, Bernard Keavney

**Affiliations:** Institute for Ageing and Health, Newcastle University, Newcastle upon Tyne, UK; Department of Cardiology, Newcastle upon Tyne Hospitals NHS Foundation Trust, Newcastle upon Tyne, UK; Department of Clinical Biochemistry, Newcastle upon Tyne Hospitals NHS Foundation Trust, Newcastle upon Tyne, UK; Institute of Genetic Medicine, Newcastle University, Newcastle upon Tyne, UK; Institute of Health and Society, Newcastle University, Newcastle upon Tyne, UK; Institute of Cardiovascular Sciences, The University of Manchester, Manchester, UK

**Keywords:** Systolic dysfunction, Diastolic dysfunction, Natriuretic peptides, Sensitivity and specificity, Aged, 80 and over

## Abstract

**Background:**

Guidelines advocate using B-type natriuretic peptides in the diagnostic work-up of suspected heart failure (HF). Their main role is to limit echocardiography rates by ruling out HF/LV dysfunction where peptide level is low. Recommended rule-out cut points vary between guidelines. The utility of B-type natriuretic peptides in the very old (85+) requires further investigation, with optimal cut points yet to be established. We examined NT-proBNP's utility, alone and in combination with history of myocardial infarction (MI), as a rule-out test for LV dysfunction in very old people with limiting dyspnoea.

**Methods:**

Design: Cross-sectional analysis.

Setting: Population-based sample; North-East England.

Participants: 155 people (aged 87-89) with limiting dyspnoea.

Measures: Dyspnoea assessed by questionnaire. Domiciliary echocardiography performed; LV systolic/diastolic function graded. NT-proBNP measured (Roche Diagnostics). Receiver operating characteristic analyses examined NT-proBNP's diagnostic accuracy for LV dysfunction.

**Results:**

AUC for LVEF less than or equal to 50% was poor (0.58, 95% CI 0.49-0.65), but good for LVEF less than or equal to 40% (0.80, 95% CI 0.73-0.86). At ESC cut point (125ng/l), few cases of systolic dysfunction were missed (NPV 94-100%, depending on severity), but echocardiography (88%) and false positive rates (56-81 per 100 screened) were high. At NICE cut point (400ng/l), echocardiography (51%) and false positive rates (33-45) were lower; exclusionary performance was good for LVEF less than or equal to 40% (1 case missed per 100 screened, 15% of cases; NPV 97%), but poor for LVEF less than or equal to 50% (16 cases missed per 100 screened, 45% of cases; NPV 68%). Incorporating isolated moderate/severe diastolic dysfunction into target condition increased the proportion of cases missed (lower NPV), whilst improving case detection. Incorporating MI history as an additional referral prompt slightly reduced the number of cases missed at expense of higher echocardiography and false positive rates.

**Conclusions:**

High echocardiography rates and poor exclusionary performance for mild degrees of systolic dysfunction and for diastolic dysfunction limit NT-proBNP's utility as a rule-out test for LV dysfunction in very old people with limiting dyspnoea. Incorporating MI history as an additional echocardiography prompt yields no overall benefit compared to using NT-proBNP level alone.

**Electronic supplementary material:**

The online version of this article (doi:10.1186/1471-2261-14-128) contains supplementary material, which is available to authorized users.

## Background

The very old (aged 85+), the most rapidly expanding age group worldwide, [1] comprise an increasing fraction of heart failure (HF) patients [2]. Early and accurate diagnosis is important as effective therapies are well established for HF with reduced left ventricular (LV) ejection fraction (HF-REF). Furthermore, there is increasing emphasis on identifying asymptomatic LV systolic dysfunction and preventing or delaying its progression to HF [3]. In older people HF diagnosis is particularly challenging due to atypical clinical presentations, [4] coupled with high levels of co-morbidity [2] which can both mimic and mask the presentation of HF. Echocardiography, the diagnostic test of choice, is expensive with limited access in many healthcare systems, [5] particularly for older people [6].

The B-type natriuretic peptides, BNP and NT-proBNP, offer a less expensive and more accessible diagnostic test for HF and LV dysfunction. Clinical guidelines advocate their use in the diagnostic work-up of suspected HF to limit the number of potential cases requiring echocardiography, by ruling out the condition where natriuretic peptide level is low, although recommended rule-out cut points vary between guidelines [7, 8]. A raised natriuretic peptide level is insufficiently specific to rule in a diagnosis, [9] with echocardiography required for further evaluation. The utility of B-type natriuretic peptides in the very old requires further investigation, and the optimal exclusionary cut points for this age group remain to be established. Natriuretic peptide levels rise with age in non-diseased individuals, [10] and with many age-related cardiac and non-cardiac morbidities [11]. Furthermore, their diagnostic accuracy is poorer in HF with preserved ejection fraction (HF-PEF), [12] which underlies around 50% of HF in people over the age of 70 [13].

We previously reported high prevalence of LV systolic and diastolic dysfunction in the very old, most cases being both symptomatic and undiagnosed [14]. We here report a prospective evaluation of the utility of NT-proBNP - alone and in combination with history of myocardial infarction (MI) [as per UK National Institute for Health and Care Excellence (NICE) Chronic HF Diagnostic Algorithm [7]] – as a rule-out test for LV systolic and diastolic dysfunction in very old people with limiting dyspnoea. Data came from the Newcastle 85+ Study, a population-based longitudinal study of health and ageing in the very old [15].

## Methods

### Participants

The recruitment strategy for the Newcastle 85+ Study has been reported [15]. In brief, people living in Newcastle or North Tyneside (North-East England) were recruited at age 85 through general practice patient lists; those living in institutions and the cognitively impaired were included. Participants were asked to undergo a cardiac phenotyping examination during their 18 or 36 month follow-up (see Supplementary appendix in Additional file 1) [14]. For this analysis, we focused on those participants who reported limiting dyspnoea, identified using a nurse-administered questionnaire. Participants were assigned to three categories: limiting dyspnoea; no limiting dyspnoea; or unclassifiable. Limiting dyspnoea indicated a clinical suspicion of HF. We did not evaluate symptoms such as orthopnoea, paroxysmal nocturnal dyspnoea, ankle oedema and fatigue; whilst some have higher specificity for HF than dyspnoea, all lack sensitivity [9]. Classical signs of HF were not evaluated being difficult to elicit in older people and lacking both sensitivity and specificity [9].

### Echocardiographic determination of LV systolic and diastolic dysfunction

Echocardiography was conducted in the home setting (own or care home) by one experienced echocardiologist who interpreted all scans. M-mode, two dimensional (2-D) and Doppler echocardiography - including tissue Doppler measurement of LV long axis velocities - was performed using a portable instrument (Vivid *i* BT06 with *i*^2^ performance package; GE Healthcare, USA). A standardised protocol was followed, conforming to guidelines from the American and British Societies of Echocardiography [16, 17] (Additional file 1: Table S1).

### NT-proBNP measurement

Plasma samples for NT-proBNP measurement were aliquoted on day of collection and stored at -80°C. NT-proBNP was measured by an electrochemiluminescent sandwich immunoassay using the Modular Analytics E170 system (Roche Diagnostics, Lewes, UK). The between-batch coefficient of variation is 1.5-3.5% from 122-4322ng/l, with an analytical range of 5-35000ng/l.

The laboratory performing the NT-proBNP assay and the echocardiologist were blinded to the echocardiographic and NT-proBNP data respectively.

### Additional data reported

Methods for additional data reported are detailed in the Supplementary Appendix (Additional file 1).

### Ethical approval

The research complied with the requirements of the Declaration of Helsinki. Ethical approval was obtained from the Newcastle and North Tyneside 1 Research Ethics Committee (reference number 06/Q0905/2). Written informed consent was obtained from participants; where people lacked capacity to consent, for example because of cognitive impairment, a formal written opinion was sought from a relative or carer.

### Statistical analysis

NT-proBNP’s distribution was non-Gaussian, therefore group comparisons were conducted on log transformed data (using t-tests). Receiver Operating Characteristic (ROC) analyses were conducted to determine NT-proBNP’s discriminatory ability for four categories of LV dysfunction (target conditions): any grade of systolic dysfunction (LVEF≤50%); moderate/severe systolic dysfunction (LVEF≤40%); a composite of either any grade of systolic dysfunction or isolated moderate/severe diastolic dysfunction; and a composite of either moderate/severe systolic dysfunction or isolated moderate/severe diastolic dysfunction. Standard errors for the area under the ROC curve (AUC) were determined using the method outlined by DeLong et al. [18]. NT-proBNP’s performance was evaluated at a range of guideline recommended and data-derived rule-out cut points. Two data-derived rule-out cut points were selected: ‘stringent’ with 95% sensitivity, chosen to miss few cases; and ‘optimised’, defined as the cut point with highest sensitivity for a specificity of at least 50%. Optimised rule-out cut points have been proposed as a means of reducing false positives whilst still limiting false negatives [19]. We report: sensitivity; specificity; positive predictive value (PPV); negative predictive value (NPV); proportion of those screened at or above the cut point i.e. in whom echocardiographic evaluation would be warranted; and numbers of cases missed and identified, and number of false positives, per 100 people screened. Analyses were carried out in Stata 12.1 (StataCorp. 2011. *Stata Statistical Software: Release 12*. College Station, TX: StataCorp LP.), with statistical significance at α = 0.05.

## Results

### Sample selection and key characteristics

**Figure 1:**
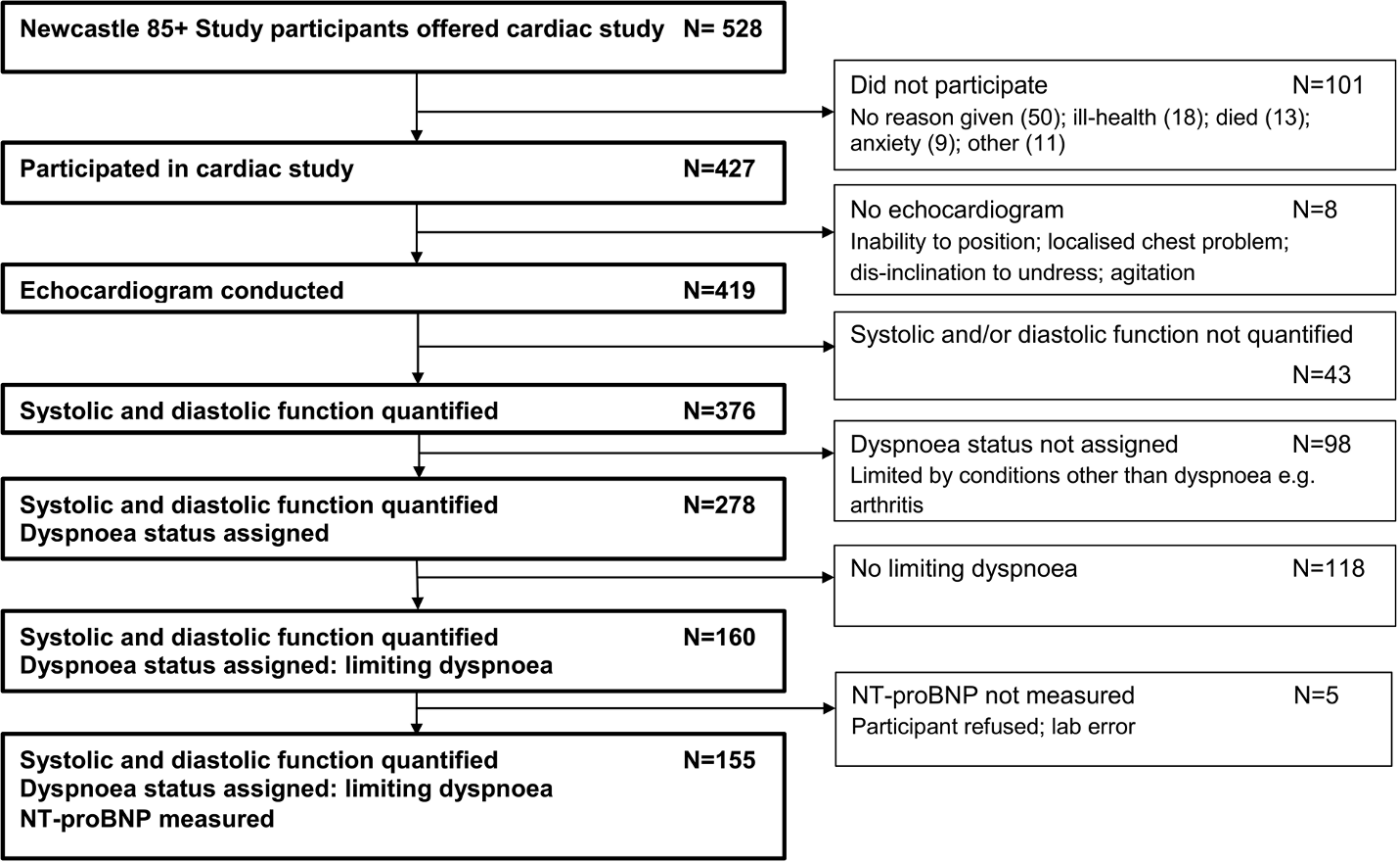
**Sample selection flow chart.**

The derivation of the analysed sample (echocardiographically-characterised participants with limiting dyspnoea and NT-proBNP data available; n=155) is shown in Figure 1 (see also Additional file 1). Table 1 summarises key demographic and clinical characteristics. The mean (standard deviation) age of participants was 88.0 (0.5) years. Systolic dysfunction (any grade, LVEF≤50%) was found in 34.2% (53/155) of dyspnoeic participants and moderate/severe systolic dysfunction (LVEF≤40%) in 8.4% (13/155); a further 19.4% (30/155) had isolated moderate/severe diastolic dysfunction. NT-proBNP ranged from 37 to 12360ng/l; median (inter-quartile range, IQR) 406 (197-1068)ng/l. NT-proBNP levels did not differ significantly between men and women (p-value=0.491). A pre-existing HF diagnosis was present in 13.6% (21/155) of dyspnoeic participants. Since we previously showed a high rate of false positive HF diagnosis in this population, [14] those participants were not excluded from the analyses. To preserve generalizability to the clinical setting in this age group where multiple conditions frequently coexist, [15] participants with other potential causes of dyspnoea were not excluded. Significant intrinsic lung disease by spirometric criteria (forced expiratory volume in one second <60% of predicted value [for age, sex and height] or forced vital capacity <70% of predicted value) was present in 14.2% (22/155) of dyspnoeic participants. Of note, natriuretic peptides have diagnostic utility for HF/LV dysfunction in patients with respiratory disease [20].

**Table 1:** Socio-demographic and clinical characteristics of participants with limiting dyspnoea (n = 155)*

Age, mean (SD); range	88.0 (0.5); 87.0-88.9
Female	65.8 (102)
Ethnic origin- white	100 (155)
Institutional care	2.6 (4)
Heart failure	13.6 (21)
Hypertension	54.2 (84)
Ischaemic heart disease	40.0 (62)
Myocardial infarction	21.9 (34)
Cerebrovascular disease	20.0 (31)
Peripheral vascular disease	3.2 (5)
Atrial fibrillation	24.5 (38)
Diabetes Mellitus	10.3 (16)
Severe renal impairment (estimated glomerular filtration rate <30 ml/min/1.73 m^2^)	3.2 (5)
Cognitive impairment (mini-mental state examination score ≤21)	3.9 (6)
Number of chronic diseases^†^, median (IQR)	5 (4-6)
Smoking Status	
Current smoker	5.2 (8)
Former regular smoker	56.5 (87)
Former occasional smoker	4.6 (7)
Never smoked	33.8 (52)
Obese (BMI >30 kg/m^2^)	11.0 (17)
On any prescribed cardiac medications	69.7 (108)
Number of prescribed cardiovascular medications, median (IQR)	1 (0-2)
Echocardiographically-characterised LV dysfunction	
Any grade of systolic dysfunction (LVEF≤50%)	34.2 (53)
Moderate/severe systolic dysfunction (LVEF≤40%)	8.4 (13)
Any grade of diastolic dysfunction	87.7 (136)
Moderate/severe diastolic dysfunction	30.3 (47)
Isolated diastolic dysfunction, any grade	58.7 (91)
Isolated moderate/severe diastolic dysfunction	19.4 (30)
NT-ProBNP, median (IQR); range (ng/l)	406 (197-1068); 37-12360

*All data are %(n) except where indicated; denominators may vary due to missing values.

^†^18 diseases considered: hypertension, ischaemic heart disease, cerebrovascular disease, peripheral vascular disease, heart failure, atrial flutter or fibrillation, arthritis, osteoporosis, chronic obstructive pulmonary disease or asthma, other respiratory disease, diabetes mellitus, hypothyroidism or hyperthyroidism, cancer diagnosed within past 5 years (excluding non-melanoma skin cancer), eye disease, dementia, Parkinson's Disease, renal impairment and anaemia.

### Comparison of NT-proBNP level across groups defined by LV function

Whilst there was notable overlap in NT-proBNP distribution across six groups defined by LV function, all dysfunction groups had significantly higher NT-proBNP than the group with no systolic dysfunction and no diastolic dysfunction graded more severe than mild (Additional file 1: Figure S1). NT-proBNP did not differ between the group with any grade of systolic dysfunction (LVEF≤50%) and the group with isolated moderate/severe diastolic dysfunction (p-value=0.761).

### ROC analyses

AUC for any grade of systolic dysfunction was poor (0.58, 95% confidence interval (CI) 0.49-0.65), whilst that for moderate/severe systolic dysfunction was good (0.80, 95% CI 0.73-0.86) (Table 2). Defining the target condition as a composite of either systolic dysfunction or isolated moderate/severe diastolic dysfunction resulted in a higher AUC for any grade of systolic dysfunction (0.64, 95% CI 0.56-0.72), but a lower AUC for moderate/severe systolic dysfunction (0.71, 95% CI 0.63-0.78).

**Table 2:** Diagnostic accuracy of NT-proBNP as rule-out test for LV dysfunction (types as specified)*

	Prevalence of specified LV dysfunction % (n)	AUC (95% CI)	NT-proBNP cut point (ng/l)	Sensitivity %	Specificity %	PPV %	NPV %	% of participants at or above cut point (echo warranted)	Number of cases picked up per 100 screened	Number of cases missed per 100 screened	Number of false positives per 100 screened
**Systolic dysfunction, any grade (LVEF≤50%)**	34.2 (53)	0.58 (0.49-0.65)									
ESC guideline rule-out cut point, 125ng/l			125	98.1	14.7	37.4	93.8	87.7	33.5	0.6	56.1
NICE guideline rule-out cut point, 400ng/l			400	54.7	50.0	36.3	68.0	51.0	18.7	15.5	32.9
Data-derived 'stringent' rule-out cut point (closest to 95% sensitivity)			131	96.2	16.7	37.5	89.5	87.7	32.9	1.3	54.8
Data-derived 'optimised' rule-out cut point (highest sensitivity with specificity at least 50%)			380	56.6	50.0	37.0	68.9	52.3	19.3	14.8	32.9
**Moderate/severe systolic dysfunction (LVEF≤40%)**	8.4 (13)	0.80 (0.73-0.86)									
ESC guideline rule-out cut point, 125ng/l			125	100.0	11.3	9.4	100.0	87.7	8.4	0.0	81.2
NICE guideline rule-out cut point, 400ng/l			400	84.6	51.4	13.8	97.3	51.0	7.1	1.3	44.5
Data-derived 'stringent' rule-out cut point (closest to 95% sensitivity)			197	100.0	26.8	11.1	100.0	75.5	8.4	0.0	67.1
Data-derived 'optimised' rule-out cut point (highest sensitivity with specificity at least 50%)			363	84.6	50.0	13.4	97.3	52.9	7.1	1.3	45.8
**Systolic dysfunction (any grade) OR isolated moderate/severe diastolic dysfunction**	53.5 (83)	0.64 (0.56-0.72)									
ESC guideline rule-out cut point, 125ng/l			125	95.2	16.7	56.8	75.0	87.7	50.9	2.6	38.7
NICE guideline rule-out cut point, 400ng/l			400	60.2	58.3	62.5	56.0	51.0	32.3	21.3	19.4
Data-derived 'stringent' rule-out cut point (closest to 95% sensitivity)			120	95.2	16.7	56.8	75.0	89.7	50.9	2.6	38.7
Data-derived 'optimised' rule-out cut point (highest sensitivity with specificity at least 50%)			319	63.9	51.4	60.2	55.2	56.8	34.2	19.4	22.6
**Moderate/severe systolic dysfunction OR isolated moderate/severe diastolic dysfunction**	27.7 (43)	0.71 (0.63-0.78)									
ESC guideline rule-out cut point, 125ng/l			125	93.0	11.6	28.8	81.3	87.7	25.8	1.9	63.9
NICE guideline rule-out cut point, 400ng/l			400	74.4	57.1	40.0	85.3	51.0	20.6	7.1	31.0
Data-derived 'stringent' rule-out cut point (closest to 95% sensitivity)			113	95.3	11.6	29.3	86.7	90.3	26.5	1.3	63.9
Data-derived 'optimised' rule-out cut point (highest sensitivity with specificity at least 50%)			298	79.1	50.0	37.8	86.2	58.1	21.9	5.8	36.1

*Abbreviations: *AUC* area under curve, *PPV* positive predictive value, *NPV* negative predictive value, *ESC* European Society of Cardiology, *NICE* National Institute for Health and Care Excellence.

Detailed legend: Diagnostic accuracy of NT-proBNP as rule-out test for LV dysfunction (types as specified) at range of guideline recommended and data-derived rule-out cut points.

Table 2 details NT-proBNP’s performance for ruling out LV dysfunction (target conditions as previously specified) using a range of guideline recommended and data-derived rule-out cut points. For the target condition of any grade of systolic dysfunction, the European Society of Cardiology (ESC) guideline rule-out cut point (for non-acute symptoms, 125ng/l) [8] missed few cases (less than one per 100 screened, 2% of all cases; NPV 94%). However, 88% of participants had NT-proBNP at or above ESC cut point, thereby warranting echocardiographic evaluation, and the false positive rate was high (56 per 100 screened). Performance at the NICE guideline rule-out cut point (400ng/l) [7] was poor; 45% of all cases would be missed (16 cases missed per 100 screened; NPV 68%), although - in comparison to the ESC cut point - fewer people would require echocardiography (51%), with a lower false positive rate (33 per 100 screened). The ESC cut point was similar to our data-derived ‘stringent’ rule-out cut point (131ng/l), whilst the NICE cut point was close to our data-derived ‘optimised’ rule-out cut point (380ng/l). For moderate/severe systolic dysfunction, at the ESC cut point no cases would be missed (NPV 100%); however, 88% of those screened would require echocardiography, with a false positive rate of 81 per 100 screened. Using the NICE cut point, one case would be missed per 100 screened (15% of all cases; NPV 97%); in comparison to the ESC cut point, fewer people would require echocardiography (51%), with a lower false positive rate (45 per 100 screened). The NICE cut point was again similar to our ‘optimised’ rule-out cut point (363ng/l), whilst the ESC cut point was somewhat lower than our ‘stringent’ cut point (197ng/l). We compared NT-proBNP’s performance for the composite target conditions of either systolic dysfunction (any grade or moderate/severe) or isolated moderate/severe diastolic dysfunction to that for the same grade of systolic dysfunction alone. Incorporating diastolic dysfunction into the target condition generally increased the proportion of all cases missed (lower NPV), whilst increasing the number of cases identified and decreasing the false positive rate (see Additional file 1: Supplementary Appendix).

We also examined test performance at two confirmatory or rule-in cut points (Additional file 1: Table S2).

### Incorporating previous MI history and NT-proBNP in risk assessment

**Figure 2:**
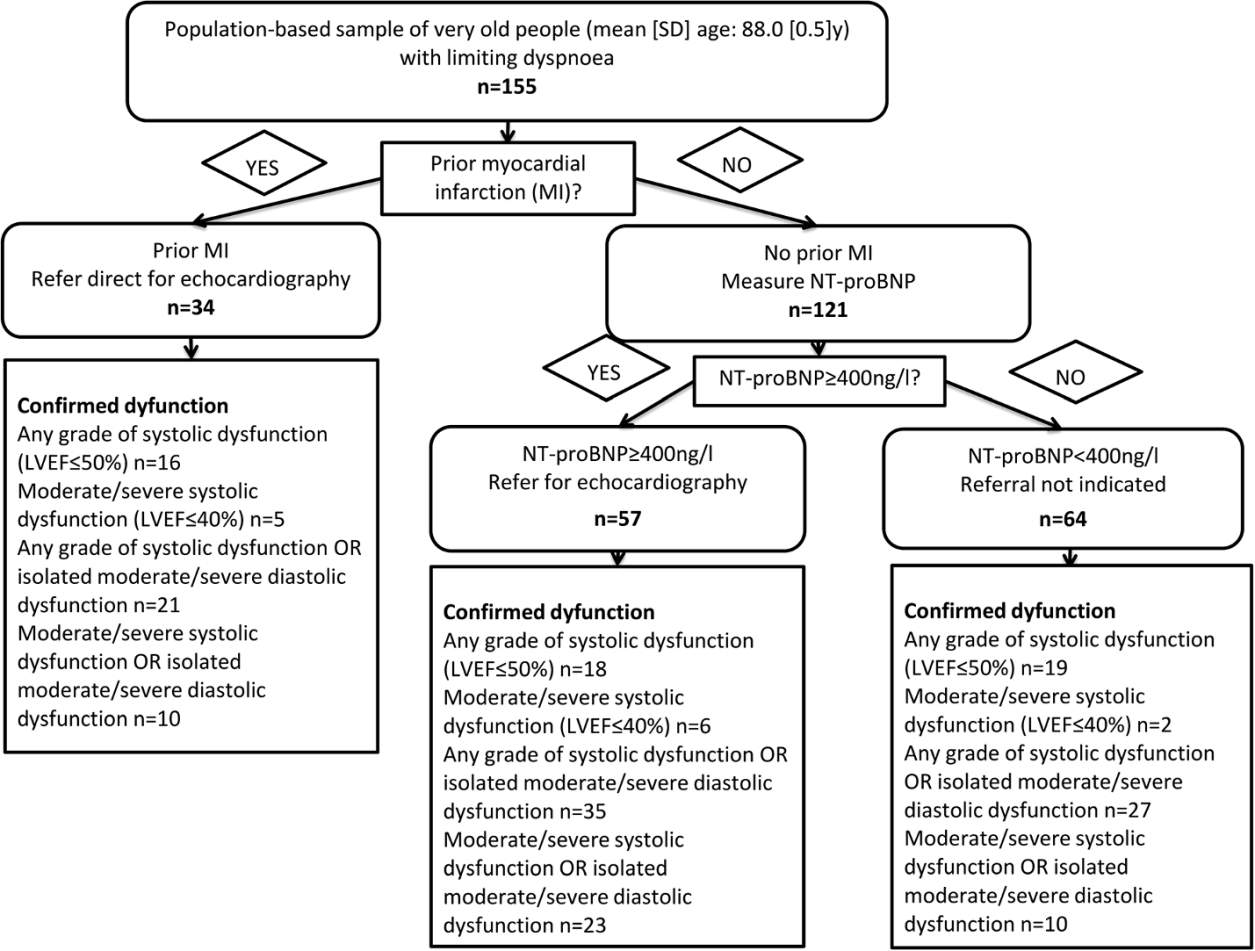
**Utility of ‘NT-proBNP or previous MI’ algorithm.** Detailed legend: Utility of ‘NT-proBNP or previous MI’ algorithm for ruling out LV dysfunction in very old people with limiting dyspnoea.

**Figure 3:**
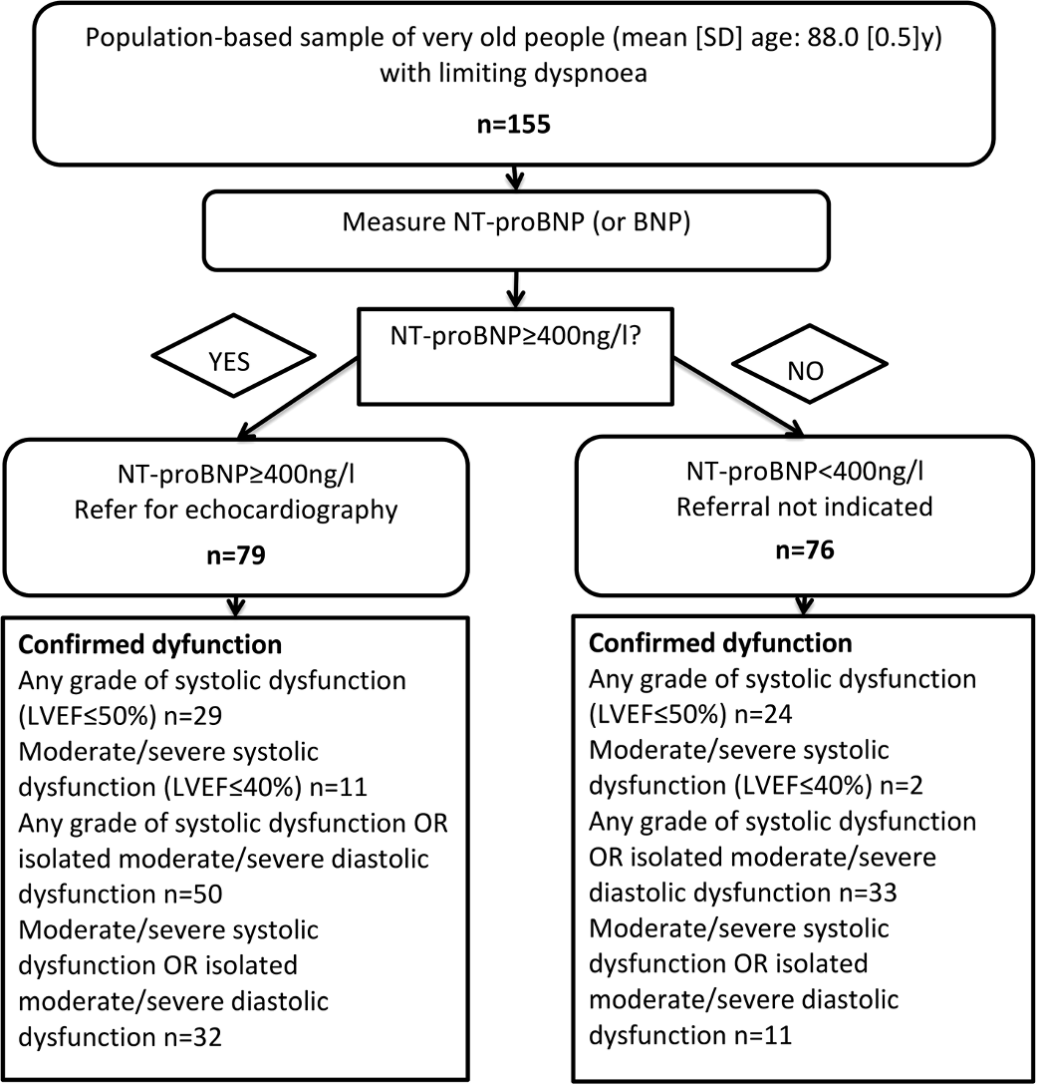
**Utility of NT-proBNP alone.** Detailed legend: Utility of NT-proBNP alone for ruling out LV dysfunction in very old people with limiting dyspnoea.

The NICE Chronic HF Diagnostic Algorithm (for use when HF is clinically suspected) recommends that individuals with a previous MI should be referred directly for echocardiographic evaluation, without preliminary natriuretic peptide measurement; those without previous MI should be referred on the basis of natriuretic peptide level [7]. We examined the algorithm’s utility for excluding LV dysfunction in our sample of very old people with limiting dyspnoea, and compared it to a strategy using NT-proBNP alone. Figure 2 presents data for the algorithm using NT-proBNP or previous MI and Figure 3 the equivalent data for the strategy using NT-proBNP alone; Table 3 summarises diagnostic accuracy. Out of our participants with limiting dyspnoea, 22% (34/155) had a previous MI. In comparison to using NT-proBNP alone, incorporating MI history resulted in modest increases in both the proportion requiring echocardiography (51% for NT-proBNP level alone versus 59% with MI incorporated) and false positive rates. The effect on the number of cases missed/identified depended on the target condition. For any grade of systolic dysfunction and the two composite conditions of either systolic dysfunction (any grade or moderate/severe) or isolated moderate/severe diastolic dysfunction, incorporating MI history slightly improved performance. In contrast, for moderate/severe systolic dysfunction the numbers of cases missed/identified remained the same.

**Table 3:** Diagnostic accuracy of NT-proBNP alone and 'NT-proBNP or previous MI' algorithm*

	Sensitivity %	Specificity %	PPV %	NPV %	% of people screened requiring echo	Number of cases picked up per 100 screened	Number of cases missed per 100 screened	Number of false positives per 100 screened
**Systolic dysfunction, any grade (LVEF≤50%)**								
NT-pro BNP alone (refer for echocardiography if ≥400ng/l)	54.7	50.0	36.3	68.0	51.0	18.7	15.5	32.9
‘NT-proBNP or previous MI' algorithm (refer for echocardiography if prior MI or NT-pro BNP ≥400ng/l)	64.2	44.1	37.4	70.3	58.7	21.9	12.3	36.8
**Moderate/severe systolic dysfunction (LVEF≤40%)**								
NT-pro BNP alone (refer for echocardiography if ≥400ng/l)	84.6	51.4	13.8	97.3	51.0	7.1	1.3	44.5
‘NT-proBNP or previous MI' algorithm (refer for echocardiography if prior MI or NT-pro BNP ≥400ng/l)	84.6	43.7	12.1	96.9	58.7	7.1	1.3	51.6
**Systolic dysfunction (any grade) OR isolated moderate/severe diastolic dysfunction**								
NT-pro BNP alone (refer for echocardiography if ≥400ng/l)	60.2	58.3	62.5	56.0	51.0	32.3	21.3	19.4
‘NT-proBNP or previous MI' algorithm (refer for echocardiography if prior MI or NT-pro BNP ≥400ng/l)	67.5	51.4	61.5	57.8	58.7	36.1	17.4	22.6
**Moderate/severe systolic dysfunction OR isolated moderate/severe diastolic dysfunction**								
NT-pro BNP alone (refer for echocardiography if ≥400ng/l)	74.4	57.1	40.0	85.3	51.0	20.6	7.1	31.0
‘NT-proBNP or previous MI' algorithm (refer for echocardiography if prior MI or NT-pro BNP ≥400ng/l)	76.7	48.2	36.3	84.4	58.7	21.3	6.5	37.4

Detailed legend: Diagnostic accuracy of 'NT-proBNP or previous MI' algorithm as rule-out test for LV dysfunction (types as specified); comparison with strategy using NT-proBNP alone.

*Abbreviations: *PPV* positive predictive value, *NPV* negative predictive value.

## Discussion

We report novel data on the utility of NT-proBNP, alone and in combination with MI history, as a rule-out test for LV systolic and diastolic dysfunction in very old people with limiting dyspnoea. The rule-out cut points recommended by the ESC (125ng/l) [8] and NICE (400ng/l) [7] guidelines differ widely, and we show that both have limitations in this age group. Focusing on NT-proBNP’s ability as a rule-out test for LV systolic dysfunction, applying the ESC cut point resulted in very few cases being missed; however, 88% of very old people with limiting dyspnoea had NT-proBNP at or above this cut point, thereby warranting echocardiography, with high false positive rates. Using the higher NICE cut point, echocardiography and false positive rates would be lower, although still substantial; however, whilst exclusionary performance was good for moderate/severe systolic dysfunction it was poor for any grade of systolic dysfunction (45% of cases missed). LVEF between 40-50% is much commoner than ≤40% (prevalence 26% and 8% respectively in our sample), and associated with prevalent and incident HF and mortality; [21] it is therefore important to identify less severe forms of dysfunction and prevent/delay their progression to more severe dysfunction and overt HF [3]. Incorporating isolated moderate/severe diastolic dysfunction (19% of our sample) into the target condition, generally increased the proportion of cases missed (lower NPV), whilst increasing the number of cases identified and decreasing the false positive rate, when compared with NT-proBNP’s performance for the same severity of systolic dysfunction alone. Although no therapy has proved effective in preventing progression of diastolic dysfunction to HF-PEF, or in improving outcomes in established HF-PEF, [8] it is important to diagnose it accurately and instigate appropriate management [22].

Through comparison with our data-derived cut points, it appears that the ESC cut point is generally an appropriate ‘stringent’ rule-out cut point (misses few cases) in the very old, whilst the NICE cut point is an appropriate ‘optimised’ rule-out cut point (cuts down false positives whilst still limiting false negatives). However, whilst these may be the most appropriate cut points for this age group, their performance metrics limit their overall utility. Interestingly, the NICE cut point has been criticised as being too high, with lower age-specific optimised rule-out cut points recommended (for NT-proBNP: <50 years, 50ng/l; 50-75 years, 75ng/l; age 75+, 223ng/l); [19] our data suggests that the NICE cut point is the appropriate optimised cut point for 87-89 year olds.

NT-proBNP’s limited diagnostic accuracy for LV dysfunction in the very old is likely to reflect the high prevalence of other cardiac and non-cardiac morbidities known to elevate natriuretic peptides, [11] compounded by the high consumption of medications (for co-morbid conditions such as hypertension) which can potentially lower peptides below threshold levels even in the presence of LV dysfunction [7]. Including MI history as an additional referral prompt (as per NICE Chronic HF Diagnostic Algorithm [7]) was of no overall benefit. It resulted in, at best, only a slight drop in cases missed (and no change for moderate/severe systolic dysfunction), at the expense of higher echocardiography and false positive rates.

Few studies have examined the diagnostic accuracy of natriuretic peptides in older people, [23] with the 85+ age group particularly under-investigated. Study setting (population-based, primary care, care home, emergency department, out-patient department etc.) affects prevalence and severity of LV dysfunction/HF and consequently test performance, and it is not possible to extrapolate findings directly from one setting to another. Our study is among the first in the very old to incorporate detailed home-based assessment of LV function with natriuretic peptide measurement, and to our knowledge is the largest population-based study of very old people with a clinical suspicion of chronic HF. Only two previous studies have focused on the symptomatic very old, both concluding that whilst natriuretic peptides have some utility as rule-out tests their performance metrics are inferior compared to younger age groups [24, 25]. Olofsson et al. examined a sample with symptoms/signs suggestive of HF from a single primary care centre (estimated n=67 aged 80+), with the emphasis on detecting systolic HF [24]. Chenevier-Gobeaux et al. investigated emergency department attendees with acute dyspnoea (n=210, aged 85+); [25] in acute studies, higher threshold natriuretic peptide values are observed in comparison to chronic dyspnoea [19]. The utility of HF diagnostic algorithms was examined by Oudejans et al. in geriatric out-patients with a clinical suspicion of new slow onset HF (n=206, aged 70-98) [26]. The performance of the NICE algorithm was superior to that found in our study, which may reflect the different target conditions studied; Oudejans et al. focused on clinical HF in contrast to our study of LV dysfunction in which natriuretic peptide performance is known to be poorer [9]. Like us, Oudejans et al. concluded that the performance of NT-proBNP alone was superior to algorithms additionally incorporating MI history.

Strengths of this study are its population-based sample, including the institutionalised and cognitively impaired, and its domiciliary echocardiographic approach incorporating assessment of both systolic and diastolic dysfunction. Hospital-based assessment of this age group is known to introduce selection bias [27]. A limitation is our use of LV dysfunction as the target condition rather than clinical HF; full clinical assessment for HF was not possible within the scope of this study. However, our focus on the use of natriuretic peptides to rule out LV dysfunction in a sample with a clinical suspicion of HF is in accordance with other important work in the field [19, 28–33]. A potential limitation is our use of limiting dyspnoea without clinical examination to define a sample with a suspicion of HF, although classical physical signs such as basal crepitations and oedema are known to lack both sensitivity and specificity in this age group [9]. Dyspnoea has a high sensitivity (89%) for chronic HF, although low specificity (51%) [9]. We did not exclude participants with other potential causes of dyspnoea as we were interested in NT-proBNP’s performance in a ‘real life’ unselected sample of dyspnoeic very old people. Furthermore, the co-existence of multiple morbidities is common in this age group [15]. Clearly the full diagnostic work-up of dyspnoeic patients should include consideration of non-cardiac conditions. The practical considerations of performing domiciliary echocardiography with a handheld instrument meant that data for some measurements was more incomplete than might have been achieved in a hospital setting [14].

## Conclusions

High echocardiography rates and poor exclusionary performance for mild degrees of systolic dysfunction and for diastolic dysfunction limit NT-proBNP’s utility as a rule-out test for LV dysfunction in very old people with limiting dyspnoea. Therefore alternative strategies merit consideration. These might include other blood-based biomarkers, either singly or in combination panels, although a recent study in a care home population found the novel biomarkers copeptin, MR-proADM and MR-proANP to have little diagnostic utility in older people with significant co-morbidity [34]. Clinical decision rules, combining natriuretic peptide measurement with additional variables (symptoms, signs and test results), have been proposed although the optimal approach and cost-effectiveness are uncertain [35]. Atypical HF presentations in the very old, [4] coupled with high levels of non-specific ECG findings and co-morbidity, [2] may limit the utility of such approaches in this age group. Optimal evaluation of this age group may require direct access to echocardiography without preliminary peptide measurement, an approach which has recently been advocated for older people with medium/high probability of HF [22]. Whilst cost-effectiveness needs to be determined, it merits further evaluation given the high costs of HF to healthcare providers [36] and rapid expansion of the very old population [1]. If this strategy were adopted, our findings imply that substantially increased echocardiography provision, in accessible settings, would be required. Community-based echocardiography services, including the option of home-based assessment, might best meet the needs of this often frail and multimorbid group.

## Electronic supplementary material

Additional file 1: **Supplementary Appendix: supplementary methods and results.** (DOCX 264 KB)

## Abbreviations

LV: Left ventricular
LVEF: Left ventricular ejection fraction
MI: Myocardial infarction
HF: Heart failure
HF-REF: Heart failure with reduced left ventricular ejection fraction
HF-PEF: Heart failure with preserved left ventricular ejection fraction
NICE: National Institute for Health and Care Excellence
ESC: European Society of Cardiology
ROC: Receiver operating characteristic
AUC: Area under the curve
NPV: Negative predictive value
PPV: Positive predictive value.
