# Supplementary material for: Utility of NT-proBNP as a rule-out test for left ventricular dysfunction in very old people with limiting dyspnoea: the Newcastle 85+ Study
Source: BMC Cardiovasc Disord. 2014 Sep 26;14:128. doi: 10.1186/1471-2261-14-128 (PMC4189162; doi:10.1186/1471-2261-14-128)
Supplement: Supplementary file 1 — Additional file 1: Supplementary Appendix: supplementary methods and results. (DOCX 264 KB) [file 12872_2014_774_MOESM1_ESM.docx]

**Utility of NT-proBNP as a rule-out test for left ventricular dysfunction in very old people with limiting dyspnoea: the Newcastle 85+ Study**

**Joanna Collerton, Andrew Kingston, Fahad Yousaf, Karen Davies, Antoinette Kenny, Dermot Neely, Carmen Martin-Ruiz, Guy MacGowan, Louise Robinson, Thomas BL Kirkwood, Bernard Keavney**

**Supplementary Appendix**

**SUPPLEMENTARY METHODS**

**Sample recruitment**

This investigation was nested in the Newcastle 85+ Study; full details of its methodology and recruitment have been reported.[1,2] In brief, members of the 1921 birth cohort living in Newcastle upon Tyne or North Tyneside (North-East England) were recruited at around age 85 using general practice patient lists as the sampling frame. The only exclusion criteria were end-stage terminal illness and behaviour that might pose a safety risk to a nurse visiting alone; people living in institutions and those with cognitive impairment were not excluded. At baseline, the Newcastle 85+ Study cohort was socio-demographically representative of the local population, and of England and Wales, including the proportion in care homes.[2] Following baseline assessment (Phase 1: 2006-7, n=854), Newcastle 85+ Study participants were re-assessed at 18 months (Phase 2: 2007-9, n=631) and again at 36 months (Phase 3: 2009-10, n=484). Loss between phases 1 and 3 was mainly due to deaths (62.7%, 232/370) with the remainder due to drop out. All Phase 2 study participants re-contacted after 1^st^ May 2008 (n=397) were eligible for a cardiac phenotyping examination, with recruitment continued into Phase 3 for those not invited in Phase 2 (n=131).

**Measurement of left ventricular (LV) function**

LV systolic function was measured using a previously validated semi-quantitative 2-D visual approach incorporating multiple echocardiographic views.[3] LV systolic dysfunction was defined by an ejection fraction (EF) cut point of 50% or less, with 40% or less defining moderate/severe dysfunction. Diastolic function was assessed as previously reported, using an approach integrating tissue Doppler imaging of the mitral valve annulus with Doppler measurements of mitral inflow.[4] Diastolic function was graded as normal or as mild (impaired relaxation), moderate (pseudo-normal filling) or severe (advanced reduction in compliance) dysfunction. We used the E/eʹ ratio, E/A ratio and mitral deceleration time (DT) to classify patients in sinus rhythm (Supplementary Table 1, upper panel). For participants in atrial fibrillation, we used the E/eʹ ratio, mitral DT and iso-volumic relaxation time (IVRT)[5] (Supplementary Table 1, lower panel).

**Supplementary Table 1: Grading scheme for diastolic function**

|  |  |  |  |  |
| --- | --- | --- | --- | --- |
| **Echo**  **parameter** | **Normal diastolic**  **function** | **Mild diastolic**  **dysfunction** | **Moderate diastolic**  **dysfunction** | **Severe diastolic**  **dysfunction** |
| *Sinus rhythm* | | | | |
| E/eʹ | <10 | <10 | ≥10 | ≥10 |
| E/A | 1-2 | <1 | 1-2 | >2 |
| Mitral DT | 150-200 | >200 | 150-200 | <150 |
| *Atrial fibrillation* | | | | |
| E/eʹ | <10 | <10 | ≥10 | ≥10 |
| Mitral DT | 150-200 | >200 | 150-200 | <150 |
| IVRT | 50-100 | >100 | 50-100 | <50 |

E/e' lateral was used except in the case of a lateral myocardial infarction where septal E/e' used

E: peak early diastolic transmitral flow velocity (cm/second)

e': early diastolic annular velocity (cm/sec)

A: peak late diastolic transmitral flow velocity (cm/second)

DT: early filling deceleration time (milliseconds)

IVRT: Iso-volumic relaxation time (milliseconds)

For participants with borderline values, or in whom the grading algorithm was inconclusive, echocardiograms were re-examined by the echocardiologist and participants either assigned to a diastolic function grade or deemed unclassifiable. Participants with paced rhythms or those in whom all data could not be acquired were considered unclassifiable. Heart rhythm was determined by contemporaneous 3 lead electrocardiogram (ECG). The cut-points for the echocardiographic parameters conformed to the British Society of Echocardiography guidelines for measurement of diastolic function.[6] We have previously demonstrated the feasibility and inter-operator/inter-reader reproducibility of this protocol in the domestic setting in this age group.[4,7] The methodological challenges of accurate measurement of left atrial volume, Valsalva manoeuvres and pulmonary venous flow (variably incorporated in diastolic function classification schemes in previous studies), precluded their use in this domiciliary study of very old people.

**Clinical diagnoses**

Pre-existing clinical diagnoses were extracted from the general practice records by a research nurse. In the UK, patients are registered with a single general practice which acts as a gatekeeper to secondary care and receives details of all hospital admissions and outpatient attendances. The review of general practice records included hospital correspondence to ensure that all pre-existing diagnoses were extracted irrespective of where the diagnosis was made. Ischaemic heart disease was determined from the following diagnoses/interventions recorded in the general practice records: angina, myocardial infarction (MI), coronary artery bypass grafts, and coronary angioplasty or coronary stent. For ischaemic heart disease and MI, participants without a diagnosis in the records were additionally assigned on the basis of the presence of relevant Minnesota codes on a 12 lead ECG; codes commencing 1-1 or 5-1 were used for ischaemic heart disease and codes commencing 1-1 for MI. Cerebrovascular disease was determined from the following diagnoses/interventions recorded in the general practice records: stroke, transient ischaemic attack and carotid endarterectomy. Diabetes mellitus was determined on the basis of a diagnosis recorded in the general practice records; participants without a diagnosis were additionally assigned on the basis of fasting blood glucose of 7mmol/l or higher.

A count of chronic diseases was calculated. Eighteen diseases were included: hypertension; ischaemic heart disease; cerebrovascular disease; peripheral vascular disease; heart failure; atrial fibrillation; arthritis; osteoporosis; chronic obstructive pulmonary disease or asthma; other respiratory disease; diabetes mellitus; thyroid disease; cancer (within last 5 years), excluding non-melanoma skin cancer; eye disease; dementia; Parkinson's Disease; anaemia; and renal impairment. For ischaemic heart disease, diabetes, and thyroid disease, presence was defined as a diagnosis either in general practice records or from electrocardiogram/blood test.[2] Atrial fibrillation was determined by 3 lead electrocardiogram, renal impairment by an estimated glomerular filtration rate of less than 30 ml/min/1.73m^2^ (Modification of Diet in Renal Disease formula[8]), and anaemia by World Health Organisation haemoglobin cut points of less than 13g/dl for men and 12g/dl for women.[9] For all other diseases, presence was taken from general practice records data alone.

For heart failure, hypertension, ischaemic heart disease, myocardial infarction, atrial fibrillation, diabetes mellitus, renal impairment and anaemia, disease status was determined at the time of the cardiac assessment with other conditions determined at the Newcastle 85+ Study baseline phase.

**Other data reported**

Contemporaneous with the cardiac assessment, data on prescribed medication was extracted from the general practice records and directly from review of participants’ medication. At the Newcastle 85+ Study baseline phase, cognitive status was assessed using the standardised mini-mental state examination;[10] body mass index calculated from measured weight and height (derived from demi-span); and ethnicity, place of residence and smoking status (current smoker, ex-smoker, never) obtained by self-report.

**SUPPLEMENTARY RESULTS**

**Sample selection**

In total, 528 Newcastle 85+ Study participants were eligible for the cardiac phenotyping examination and 80.9% (427/528) took part; echocardiography was conducted in 419 of these. LV systolic function could be quantified by semi-quantitative 2-D visual estimate in 95.0% (398/419) of those undergoing echocardiography, and LV diastolic function was classifiable in 92.1% (386/419). Both systolic and diastolic LV function was quantified in 89.7% (376/419) of participants undergoing echocardiography.[4] Classifiable dyspnoea data was available in 73.9% (278/376) of echocardiographically-characterised participants. Dyspnoea status could not be assigned in 98 participants, chiefly due to their uncertainty as to whether dyspnoea limited their activity owing to the presence of other limiting conditions (e.g. arthritis). Levels of echocardiographically assessed LV dysfunction, pre-existing HF diagnoses and NT-proBNP were broadly comparable between those with and without classifiable dyspnoea data. Of the 278 echocardiographically-characterised participants with classifiable dyspnoea data, 57.6% (160/278) reported limiting dyspnoea. NT-proBNP data was available for 155 (96.9%) of these, who formed the sample for the principal analyses.

**Supplementary Figure 1: Comparison of NT-proBNP level across six groups defined by LV function**

**
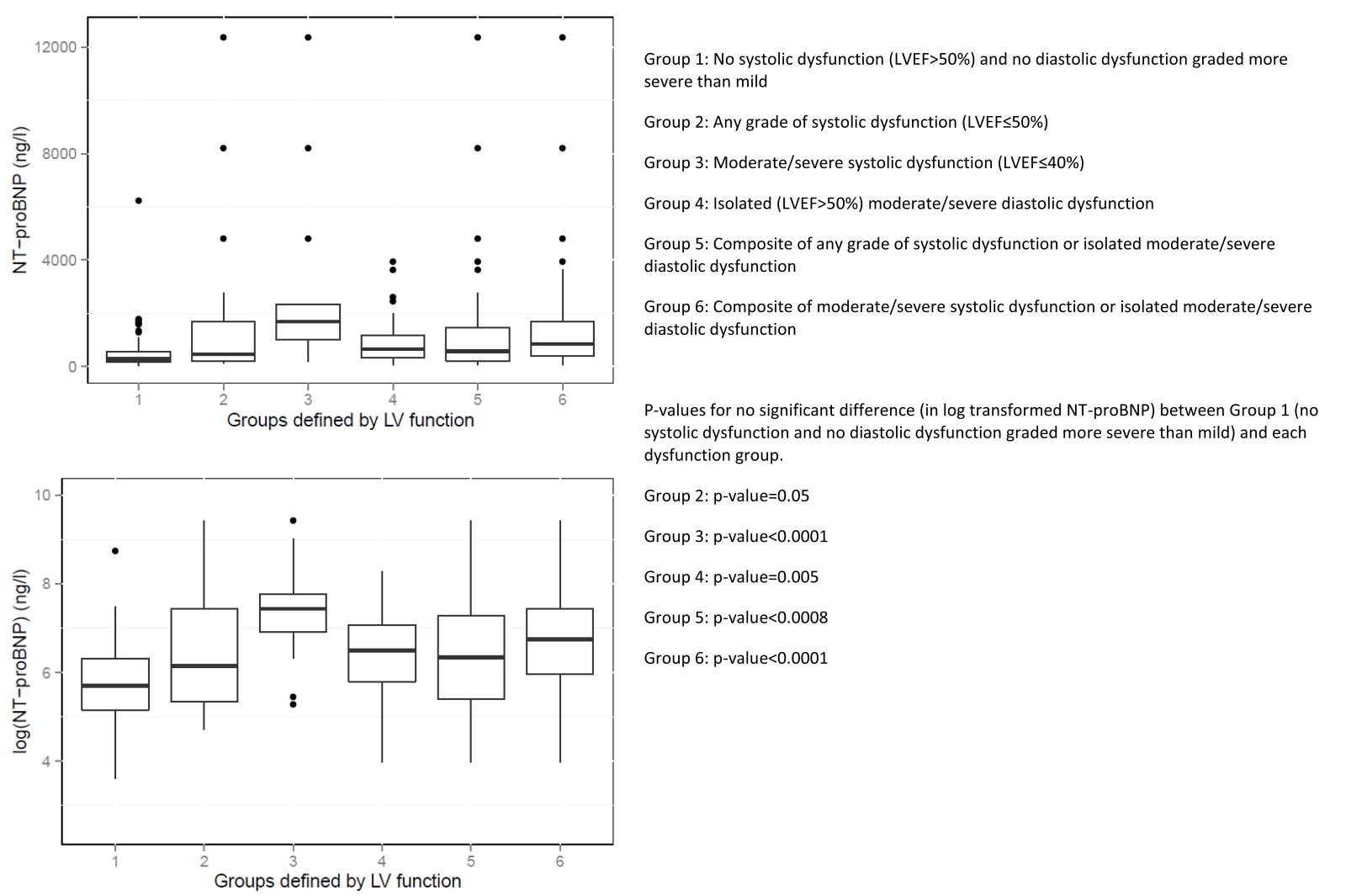
**

**NT-proBNP’s performance as a rule-out test for the composite target conditions of either systolic dysfunction (any grade or moderate/severe) or isolated moderate/severe diastolic dysfunction**

We compared NT-proBNP’s performance for the composite target condition of either systolic dysfunction (any grade) or isolated moderate/severe diastolic dysfunction to that for systolic dysfunction (any grade) alone. Using the ESC cut point (125ng/l), in comparison to the target condition of systolic dysfunction alone, the proportion of cases missed increased (5% versus 2%), whilst the number of cases identified increased (51 versus 34 per 100 screened) and the false positive rate decreased (39 versus 56 per 100 screened); the proportion requiring echocardiography was high (88%). At the NICE cut point (400ng/l) performance was poor although, in comparison to systolic dysfunction alone, a slightly lower proportion of cases were missed (40% versus 45%) and the number of cases identified increased (32 versus 19 per 100 screened), whilst the false positive rate decreased (19 versus 33 per 100 screened); the proportion requiring echocardiography was 51%. The ESC cut point was almost identical to our data-derived ‘stringent’ rule-out cut point (120pg/ml); the NICE cut point was somewhat higher than our data-derived ‘optimised’ rule-out cut point (319pg/ml).

For the composite target condition of either moderate/severe systolic dysfunction or isolated moderate/severe diastolic dysfunction, compared to the target condition of moderate/severe systolic dysfunction alone, at the ESC cut point the proportion of cases missed increased (7% versus 0%) whilst the number of cases identified increased (26 versus 8 per 100 screened) and the false positive rate decreased (64 versus 81 per 100 screened); 88% would require echocardiography. At the NICE cut point, compared to the target condition of moderate/severe systolic dysfunction alone, a higher proportion of cases were missed (26% versus 15%) whilst the number of cases identified increased (21 versus 7 per 100 screened) and the false positive rate decreased (31 versus 45 per 100 screened); the proportion requiring echocardiography was 51%. The ESC cut point was again similar to our data-derived ‘stringent’ rule-out cut point (113pg/ml); the NICE cut point was somewhat higher than our data-derived ‘optimised’ rule-out cut point (298pg/ml).

**Confirming LV dysfunction: NT-proBNP's performance at selected rule-in cut points**

We examined test performance at two confirmatory or rule-in cut points: the NICE cut point of 2000ng/l, above which urgent (within 2 weeks) echocardiographic evaluation is advised; and a data-derived rule-in cut point with 95% specificity (Supplementary Table 2). Our data-derived rule-in cut points (1755-2348ng/l, depending on target condition) were broadly similar to the NICE cut point. Using the NICE cut point, 8% of very old people with limiting dyspnoea would require urgent echocardiography with a low false positive rate (1-6 per 100 screened, depending on target condition); however, few cases would be identified (3-8 per 100 screened) with 69-87% of all cases missed (NPV 50-94%).

**Supplementary Table 2: Diagnostic accuracy of NT-proBNP as a rule-in test for LV dysfunction (types as specified) at guideline recommended and data-derived rule-in cut points**

|  |  | **Prevalence of specified LV dysfunction % (n)** | **AUC (95% CI)** | **NT-proBNP cut point (ng/l)** | **Sensitivity %** | **Specificity %** | **PPV %** | **NPV %** | **% of participants at or above cut point (echo warranted)** | **Number of cases picked up per 100 screened** | **Number of cases missed per 100 screened** | **Number of false positives per 100 screened** |
| --- | --- | --- | --- | --- | --- | --- | --- | --- | --- | --- | --- | --- |
| ***Systolic dysfunction, any grade (LVEF≤50%)*** | | 34.2 (53) | 0.58 (0.49-0.65) |  |  |  |  |  |  |  |  |  |
|  | NICE guideline cut point indicating need for urgent assessment (>2000ng/l) | |  | 2000 | 13.2 | 94.1 | 53.8 | 67.6 | 8.4 | 4.5 | 29.7 | 3.9 |
|  | Data-derived rule-in cut point (closest to 95% specificity) |  |  | 2250 | 13.2 | 95.1 | 58.3 | 67.8 | 7.7 | 4.5 | 29.7 | 3.2 |
| ***Moderate/severe systolic dysfunction (LVEF≤40%)*** | | 8.4 (13) | 0.80 (0.73-0.86) |  |  |  |  |  |  |  |  |  |
|  | NICE guideline cut point indicating need for urgent assessment (>2000ng/l) |  |  | 2000 | 30.8 | 93.7 | 30.8 | 93.7 | 8.4 | 2.6 | 5.8 | 5.8 |
|  | Data-derived rule-in cut point (closest to 95% specificity) |  |  | 2348 | 30.8 | 95.1 | 36.4 | 93.8 | 7.1 | 2.6 | 5.8 | 4.5 |
| ***Systolic dysfunction (any grade) OR isolated moderate/severe diastolic dysfunction*** | | 53.5 (83) | 0.64 (0.56-0.72) |  |  |  |  |  |  |  |  |  |
|  | NICE guideline cut point indicating need for urgent assessment (>2000ng/l) |  |  | 2000 | 14.5 | 98.6 | 92.3 | 50.0 | 8.4 | 7.7 | 45.8 | 0.6 |
|  | Data-derived rule-in cut point (closest to 95% specificity) |  |  | 1755 | 18.1 | 95.8 | 83.3 | 50.4 | 11.6 | 9.7 | 43.8 | 1.9 |
| ***Moderate/severe systolic dysfunction OR isolated moderate/severe diastolic dysfunction*** | | 27.7 (43) | 0.71 (0.63-0.78) |  |  |  |  |  |  |  |  |  |
|  | NICE guideline cut point indicating need for urgent assessment (>2000ng/l) |  |  | 2000 | 20.9 | 96.4 | 69.2 | 76.1 | 8.4 | 5.8 | 21.9 | 2.6 |
|  | Data-derived rule-in cut point (closest to 95% specificity) |  |  | 1917 | 23.3 | 95.5 | 66.7 | 76.4 | 9.7 | 6.5 | 21.3 | 3.2 |

**Abbreviations**: AUC, area under curve; PPV, positive predictive value; NPV, negative predictive value; NICE, National Institute for Health and Care Excellence

**REFERENCES**

1. Collerton J, Barrass K, Bond J, Eccles M, Jagger C, James O, Martin-Ruiz C, Robinson L, von Zglinicki T, Kirkwood T: **The Newcastle 85+ study: biological, clinical and psychosocial factors associated with healthy ageing: study protocol**. *BMC Geriatr* 2007, **7**:14.

2. Collerton J, Davies K, Jagger C, Kingston A, Bond J, Eccles MP, Robinson LA, Martin-Ruiz C, von Zglinicki T, James OFW*,* Kirkwood TBL: **Health and disease in 85 year olds: baseline findings from the Newcastle 85+ cohort study**. *BMJ* 2009, **339**:b4904.

3. Redfield MM, Jacobsen SJ, Burnett JC, Jr., Mahoney DW, Bailey KR, Rodeheffer RJ: **Burden of systolic and diastolic ventricular dysfunction in the community: appreciating the scope of the heart failure epidemic**. *JAMA* 2003, **289**(2):194-202.

4. Yousaf F, Collerton J, Kingston A, Kenny A, Davies K, Jagger C, Robinson L, Kirkwood TB, Keavney B: **Prevalence of left ventricular dysfunction in a UK community sample of very old people: the Newcastle 85+ study**. *Heart* 2012, **98**(19):1418-1423.

5. Al-Omari MA, Finstuen J, Appleton CP, Barnes ME, Tsang TSM: **Echocardiographic assessment of left ventricular diastolic function and filling pressure in atrial fibrillation**. *Am J Cardiol* 2008, **101**(12):1759-1765.

6. British Society of Echocardiography Education Committee (Masani N, Wharton G, Allen J, Chambers J, Graham J, Jones R, Rana B, Steeds R) **Echocardiography: Guidelines for Chamber Quantification**. 2010.

7. Collerton J, Martin-Ruiz C, Kenny A, Barrass K, von Zglinicki T, Kirkwood T, Keavney B, Newcastle 85+ Core Study Team: **Telomere length is associated with left ventricular function in the oldest old: the Newcastle 85+ study**. *Eur Heart J* 2007, **28**(2):172-176.

8. Manjunath G, Sarnak MJ, Levey AS: **Prediction equations to estimate glomerular filtration rate: an update**. *Curr Opin Nephrol Hypertens* 2001, **10**(6):785-792.

9. de Benoist B, McLean E, Egli I, Cogswell M. **Worldwide prevalence of anaemia 1993-2005. WHO global database on anaemia** [http://www.who.int/vmnis/anaemia/prevalence/en/index.html]

10. Molloy DW, Standish TI: **A guide to the standardized Mini-Mental State Examination**. *Int Psychogeriatr* 1997, **9 Suppl 1**:87-94. .
